# Relationship between Marriage and Prediabetes among Healthcare Workers: Mediating Effect of Triglycerides

**DOI:** 10.3390/medicina60091418

**Published:** 2024-08-30

**Authors:** Yong-Hsin Chen, Jia-June Lin, Hsiu-Mei Tang, Ching-Wen Yang, Gwo-Ping Jong, Yi-Sun Yang

**Affiliations:** 1 The Department of Health Policy and Management, Chung Shan Medical University, Taichung 402, Taiwan; a6328539@gmail.com; 2 Department of Public Health, Chung Shan Medical University, Taichung 402, Taiwan; 3 Department of Occupational Safety and Health, Chung Shan Medical University Hospital, Taichung 402, Taiwan; tang.hm76@gmail.com (H.-M.T.); thesarina1120@gmail.com (C.-W.Y.); 4 Nursing Department, Chung Shan Medical University Hospital, Taichung 402, Taiwan; cshy1506@csh.org.tw; 5 Department of Internal Medicine, Chung Shan Medical University Hospital, Taichung 402, Taiwan; 6 Institute of Medicine, Chung Shan Medical University, Taichung 402, Taiwan

**Keywords:** diabetes, prediabetes, impaired fasting glucose, triglycerides, marriage, obesity

## Abstract

*Background and Objectives*: In the literature, relationships between being married and having prediabetes or diabetes are inconsistent. We aimed to investigate whether marriage is a protective or risk factor for prediabetes and to uncover new insights into its impact on prediabetes. *Materials and Methods*: In this cross-sectional observational study, questionnaires were distributed by email to 1039 staff members who participated in an employee health check from a hospital affiliated with a medical university in Taiwan. Fasting blood glucose and triglyceride (TG) levels were checked and the questionnaires elicited basic demographic characteristics and included the Copenhagen Burnout Inventory and Nordic Musculoskeletal Questionnaire. The chi-square test or Fisher’s exact test, logistic regression, and mediation analysis were conducted for statistical analysis. *Results*: Among the group aged 20–37 years, married (OR = 1.89, 95%CI: 1.08, 3.33), obesity (OR = 2.95, 95%CI: 1.49, 5.83), neck and shoulder pain (OR = 1.31, 95%CI: 1.01, 1.69), and elevated TG levels (OR = 1.01, 95%CI: 1.00, 1.01) were independent risk factors for prediabetes (impaired fasting glucose). For those >38 years old, overweight (OR = 2.08, 95%CI: 1.27, 3.43), obesity (OR = 4.30, 95%CI: 2.38, 7.79), and elevated triglyceride (TG) (OR = 1.003, 95%CI: 1.00, 1.01) were the independent risk factors for impaired fasting glucose. Increased TG levels serve as a mediating factor (Z_m_ = 2.64, *p* < 0.01) linking marriage to an increased risk of prediabetes for the group aged 20–37 years. *Conclusions*: TGs play a significant role in the association between marriage and prediabetes among the group aged 20–37 years. Therefore, dietary habits, especially those of young adult couples should be considered. Our findings connect marital status to prediabetes, facilitating advances in diabetes prevention.

## 1. Introduction

Diabetes is a chronic condition that leads to premature death and an economic burden on the healthcare system, with 46.6% of deaths in adults aged < 60 years [1]. Per the Health Promotion Administration, Ministry of Health and Welfare in Taiwan, Taiwan has >2 million diabetic patients, increasing by 25,000 per year. Of individuals with prediabetes (i.e., having glycemic variables higher than normal but lower than diabetes thresholds), approximately 5–10% develop diabetes per year [2]. Fortunately, restoring normoglycemia during prediabetes or very early type 2 diabetes can prevent diabetes progression [3]. Despite their medical expertise, healthcare workers may neglect their own health due to busy clinical work, increasing their diabetes risk. Thus, research on diabetes prevention among them is necessary.

Research suggests that chronic pain and body weight are associated with diabetes/prediabetes [4]. For instance, individuals with diabetes/prediabetes have a high prevalence of chronic pain symptoms (e.g., lower limb, back, and neck pain) [4]. Obesity was associated with a greater risk of type 2 diabetes [5]. The prevalence of prediabetes was significantly greater in adolescents and young adults with obesity than in those with normal weight [6]. A study of men and women aged ≥ 18 years in South Korea suggested that long working hours increased the risk of developing diabetes in prediabetes patients [7]. For mental health and diabetes, a systematic review and meta-analysis suggested that burnout is associated with an increased risk of type 2 diabetes [8]. For workstyle, night shifts and rotating shifts were associated with an increased risk of type 2 diabetes [9].

Research has also suggested that lifestyle interventions such as diet or exercise effectively reduce the progression from prediabetes to diabetes [10,11]. One study showed poor diet as a main risk factor for diabetes [12]. Becoming married (or living as married) can change eating habits [13], possibly due to women’s dinner choices being often limited by their husbands’ preferences for meat, vegetables, and a smaller variety of foods than they prefer [14]. Another study supported the aforementioned view that compared with women who were married or single, women eat more meat and snacks and drink higher-fat milk and more alcohol when married or cohabitating with a man. Conversely, men tended to eat less meat, drink lower-fat milk, and use less alcohol after marriage than before marriage [15]. Eating together after marriage might also affect the development of diabetes in spouses. For instance, a large prospective biracial cohort and meta-analysis showed a positive association between spousal diabetes status and diabetes development [16]. Research has suggested that spouses are less likely to have diabetes than those who are unmarried, widowed, or divorced [17]. However, another study showed less risk of type 2 diabetes in widowed women than in married women [18]. An exploratory cross-sectional survey of females showed that being married significantly increased the risk for prediabetes and diabetes [19]. These results are inconsistent. Thus, to provide insights into prediabetes development, we explored whether marriage is a protective or risk factor for prediabetes.

Triglycerides (TGs) are a risk factor for type 2 diabetes [20] and are the main lipid component of dietary fat, animal fat [21], and milk lipids (>98%) [22]. In overnutrition, increased fatty acids in the liver lead to higher blood fatty acid levels and more hepatic TG storage [23]. Excess TG is packaged into chylomicrons, enters the lymphatic system, reaches the plasma, and is absorbed by muscle and adipose tissue [24]. Thus, TG is closely related to fat intake. The previous study demonstrated elevated TG levels might be closely associated with changes in diet habits [25]. Thus, we posed two hypotheses (H1 and H2) to verify whether TG change is a primary cause of increased prediabetes risk during marriage.

**H1:**  *Marrying is an independent risk factor for prediabetes.*

**H2:**  *Increased TG is a mediating factor that increases prediabetes risk during marriage.*

## 2. Materials and Methods

To conduct this cross-sectional observational study, we distributed QR code-linked questionnaires by email in March 2023 to 2369 staff members from a hospital affiliated with a medical university in Taichung, Taiwan, who were participating in an employee health check. We received 1630 responses; 1039 were deemed valid exclusion criteria were missing data, no health check report, a history of diabetes, or fasting blood sugar (FBG) over 125 mg/dL. Participants’ demographic/living/work data (e.g., family structure, living habits, occupation, physical health) were collected using questionnaires. Because burnout [8] and musculoskeletal pain [4] are closely related to prediabetes and diabetes, the Copenhagen Burnout Inventory and the Nordic Musculoskeletal Questionnaire (NMQ) were used to measure those of the participants.

### 2.1. Burnout Measurement

The Copenhagen Burnout Inventory has very high internal reliability and is suitable for use in the human services sector; it includes a personal burnout (PB) scale, a work-related burnout scale, and a client burnout scale, measuring the dimensions of burnout separately [26]. For the purposes of suitability for participants in all different professional fields, we adopted the PB scale, which is a generic scale, to make sure that we were able to compare individuals regardless of occupational status [26]. The first six questions pertained to PB:

1. How often do you feel tired?
2. How often are you physically exhausted?
3. How often are you emotionally exhausted?
4. How often do you think “I can’t take it anymore?”
5. How often do you feel worn out?
6. How often do you feel weak and susceptible to illness?

Response options were always, often, sometimes, seldom, and never/almost never, with scores of 100, 75, 50, 25, and 0, respectively. The mean score of the six items represented each participant’s burnout level.

### 2.2. Musculoskeletal Pain Measurement

We also used the NMQ to measure the presence of pain attributable to work-related factors in the preceding year, which is a repeatable, sensitive, and reliable measurement measured of pain [27,28,29]. The response options for the presence of pain sites were the neck, both shoulder, upper back, waist or lower back, both elbows, both wrists, both hip/thigh/buttocks, both knees, and both ankles, respectively. The options for the frequency of each pain site were every day, once per week, once per month, once per half a year, and at least once every half a year (relatively scored as 100, 80, 60, 40, and 20 points). The options for degree of seriousness were “even life is affected”, “need to take leave to recuperate”, “work ability is significantly reduced”, “slightly reduce work capacity”, and “does not affect life and work at all” (relatively scored as 100, 75, 50, 25, and 5 points). According to the frequency score multiplied by the seriousness degree score, we used factor analysis [30] to determine new underlying variables to effectively explain the questionnaire; the new variables identified by the new factor loadings are listed and marked in Supplementary Information Table S1.

### 2.3. Demographic/Living/Work Data

The response options for sex on the questionnaire were female or male. Age was filled out by participants. Married and others were the response options for marital status. Yes and No were the response options for whether the participant raises children and whether he or she lives with his or her parents, respectively. Never, occasionally, one cup every day, two cups per day, and at least two cups per day were the response options for drinking coffee. The response options for alcohol use habits in the past month were never, occasionally, and drinking every day. In addition, daily sleeping time was surveyed, and the response options were <5 h, 5–6 h, 6–7 h, 7–8 h, and >8 h. We also surveyed participants’ exercise habits. The response options were never, less than one time monthly, at least more than one time monthly, at least more than one time weekly, and at least one time daily, with scores of 100, 75, 50, 25, and 0 points, respectively.

Height and weight were recorded and classified per the definitions of underweight [body mass index (BMI) < 18.5], healthy weight (18.5 ≤ BMI < 24.0), overweight (24.0 ≤ BMI < 27.0), and obese (BMI ≥ 27.0) by the Health Promotion Administration, Ministry of Health and Welfare, Taiwan. Chronic diseases were prelisted in the questionnaire and ticked by the participants. One or more diseases indicated the category “suffering from chronic disease”. The response options for professional field were nurses, administration staff, physicians, including attending physicians, residents and nurse practitioners, and technical staff. The daily work time (hours) was self-reported. For shift work, response options were irregular, regular, night, and day shift work. Response options for education degree were PhD, master, bachelor, and others.

### 2.4. Health Check Data

FBG and TG levels were checked and confirmed by the laboratory of a hospital affiliated with a medical university. In 2003, the American Diabetes Association issued new impaired fasting glycemia diagnostic criteria, widening the FBG range from 110–125 to 100–125 mg/dL [31]. Thus, we used the new criteria (100–125 mg/dL) to define prediabetes. Because the criteria fit FBG, prediabetes is called impaired fasting glucose (IFG) hereafter.

### 2.5. Statistic Methods

We used three steps to confirm H1 and H2:

Step 1: The chi-square test or Fisher’s exact test was used to determine confounders of IFG for every stratified age group, owing to the close relationship between age and diabetes.

Step 2: A logistic regression model was established for the correlation between marital status and the IFG risk in a stratified analysis of age, confirming marital status as an independent risk factor for increased risk of IFG.

Step 3: Mediation models were used to confirm whether increased TG is a mediating factor between marriage status and IFG. The mediating effect was assessed using the strategy of Baron and Kenny [32], in which (1) the independent variable significantly affects the mediating factor (the first-stage effect), (2) the independent variable significantly affects the dependent variable in the absence of the mediating factor, (3) the mediating factor has a significant unique effect on the dependent variable (the second-stage effect), and (4) the effect of the independent variable on the dependent variable (the direct effect) weakens upon the addition of a mediating factor to the model. If the mediation factor or dependent variable is a categorical variable, the original formula of the Sobel test is rederived into a new formula per Iacobucci (2012) [33]:

Z_(mediation) (Z_m) = (a/s_a × b/s_b)/√(〖(a/s_a)〗^2^ + (〖b/s_b)〗^2^ + 1)

First-stage effect: a is the linear or logistic regression coefficient of the independent variable against the mediating factor;

Second-stage effect: b is the regression coefficient of the mediating factor against the dependent variable in the linear or logistic regression model;

where s_a and s_b are the standard deviations of a and b, respectively. Z_m values exceeding |1.96|, |2.57|, and |3.90| (for a two-tailed test) are significant at α = 0.05, 0.01, and 0.0001, respectively.

SAS Enterprise Guide 7.1 software (SAS Institute Inc., Cary, NC, USA) was used for analysis; significance was set at *p* < 0.05.

## 3. Results

### 3.1. Description of Survey Variables and Prediabetes for Participants

Table 1 describes the survey and demographic variables for 1039 female-dominated health workers (85.18%): average age, 37.50 ± 9.95 years; 43.60% married, 38.02% raising children; 59.29%, 18.48%, and 13.76% were healthy weight, overweight, and obese, respectively; 45.72% nurses; 11.65% physicians; average daily work time, 8.52 ± 0.88 h per participant; and 27.91% reported recent shift work (i.e., irregular and regular). Notably, 220 participants (21.17%) fulfilled the prediabetes criteria, per FBG. For all participants, the mean FBG and TG levels were 93.49 ± 8.60 and 74.03 ± 53.63 mg/dL, respectively.

Regarding prediabetes, males had a greater prevalence of IFG than females did (23.46–38.36%, *p* < 0.05). IFG prevalence was greater for married than not married, with no age stratification (28.26% vs. 15.70%, *p* < 0.001). Only married participants aged 20–37 years had a greater risk of IFG than their unmarried counterparts (24.11% vs. 12.59%, *p* = 0.004). Participants raising children had a greater risk of developing IFG than those not raising children, with no age stratification (27.34 vs. 17.39%, *p* < 0.001); however, only participants aged 20–37 years and raising children had a greater risk of developing IFG than did those not raising children in age stratification (25.61 vs. 13.08%, *p* = 0.007). Despite no association between coffee drinking frequency and IFG (*p* = 0.111), we detected a dose–response relationship between increased frequency and increased risk (never–at least two cups per day, 18.34–35.71%). For body weight, obese or overweight participants had a greater risk of IFG than healthy or underweight participants, regardless of age (for all *p* < 0.001). The proportion of obese participants with IFG in the group aged > 38 years was >50% (52.94%). Prediabetes risk in participants with chronic diseases (excluding diabetes) was greater than that for all participants without chronic diseases (25.45 vs. 18.62%, *p* = 0.009). IFG risk varied significantly among professions with no age stratification (*p* = 0.005): Nurses were the lowest (17.05%), and administrative staff and others were the highest (26.69%). IFG risk only differed among professions in the >38 years group (*p* = 0.043) and was highest for administrative staff and others (34.57%). With no age stratification, IFG risk differed among the shift work types (*p* = 0.008), with the highest risk for the day shift (24.68%).

Education level was associated with IFG risk with no age stratification and for the group aged 20–37 years (*p* = 0.013; 0.004). The proportion of participants with IFG who had a PhD was the highest (44.44%), followed by those with a master’s degree (24.09%) and bachelor’s degree (19.74%). Among the group aged 20–37 years, for those with IFG, 18.37% and 14.05% had a master’s degree and a bachelor’s degree, respectively.

The mean frequency coefficients of pain in the neck and shoulder and the ankle were determined using factor analysis with no age stratification for those aged 20–37 years and aged > 38 years (Table 1).

Mean FBG was 93.49 ± 8.60 mg/dL for no age stratification and 91.89 ± 7.62 mg/dL and 95.18 ± 9.25 mg/dL for the groups aged 20–37 and 38 years, respectively. The mean TG concentration was 74.03 ± 53.63 mg/dL for no age stratification and 65.82 ± 48.36 mg/dL and 82.68 ± 57.47 mg/dL for the groups aged 20–37 and years, respectively.

### 3.2. A Logistic Regression Model of Marital Status and IFG

Table 2 shows the simple (M_0_) and multiple (M_1_) logistic regression models for prediabetes by age. For the M_0_ model with no age stratification, female (OR = 0.55, 95% CI: 0.38, 0.81), aged > 38 years (OR = 2.17, 95% CI: 1.59, 2.95), married (OR = 2.12, 95% CI: 1.56, 2.86), raising children (OR = 1.79, 95% CI: 1.32, 2.42), drinking coffee every day (OR = 1.47, 95% CI: 1.09, 1.99), overweight (OR = 2.15, 95% CI: 1.47, 3.14), obese (OR = 3.98, 95% CI: 2.68, 5.91), chronic diseases (excluding diabetes) (OR = 1.49, 95% CI: 1.10, 2.02), nurses (OR = 0.57, 95% CI: 0.40, 0.79), neck and shoulder pain (OR = 1.23, 95% CI: 1.07, 1.43), and higher TG (OR = 1.01, 95% CI: 1.006, 1.011) were confounders of IFG risk. Because of possible collinearity concerns between being married and raising children, we excluded the dummy variable of raising children from the regression models. The confounders of IFG were adjusted variables in the M_1_ models. For all participants, the M_1_ model showed that marital status (OR = 1.65, 95% CI: 1.14, 2.38), overweight (OR = 1.66, 95% CI: 1.10, 2.50), obesity (OR = 3.45, 95% CI: 2.22, 5.37), neck and shoulder pain (OR = 1.19, OR = 1.02, 1.40), and higher TG (OR = 1.004, 95% CI: 1.00, 1.01) were independent risk factors for IFG.

The M_0_ model for the group aged 20–37 years showed females (OR = 0.51, 95% CI: 0.29, 0.91), married (OR = 2.21, 95% CI: 1.31, 3.71), raising children (OR = 2.29, 95% CI: 1.30, 4.03), sleeping for less than 6 h (OR = 1.68, 95% CI: 1.04, 2.71), obesity (OR = 3.70, 95% CI: 2.06, 6.65), neck and shoulder pain (OR = 1.30, 95% CI: 1.03, 1.65), and TG (OR = 1.01, 95% CI: 1.005, 1.015) were confounders of IFG risk. The M_1_ model for the group aged 20–37 years showed that marital status (OR = 1.89, 95% CI: 1.08, 3.33), obesity (OR = 2.95, 95% CI: 1.49, 5.83), neck and shoulder pain (OR = 1.31, 95% CI: 1.01, 1.69), and TG (OR = 1.01, 95% CI: 1.00, 1.01) were independent risk factors for IFG.

The M_0_ model for the group > 38 years of age showed that female (OR = 0.56, 95% CI: 0.33, 0.94), exercising at least once weekly (OR = 1.51, 95), overweight (OR = 2.28, 95% CI: 1.43, 3.63), obese (OR = 4.54, 95% CI: 2.61, 7.91), nurse (OR = 0.57, 95% CI: 0.37, 0.89), technical staff member (OR = 0.47, 95% CI: 0.23, 0.98), and higher TG (OR = 1.01, 95% CI: 1.003, 1.01) were confounders of IFG. The M_1_ model for the group >38 years of age showed that overweight (OR = 2.08, 95% CI: 1.27, 3.43), obese (OR = 4.30, 95% CI: 2.38, 7.79), and higher TG (OR = 1.003, 95% CI: 1.00–1.01) were the only independent risk factors for IFG.

### 3.3. Mediation Models of Marriage State, TG, and IFG

**Figure 1:**
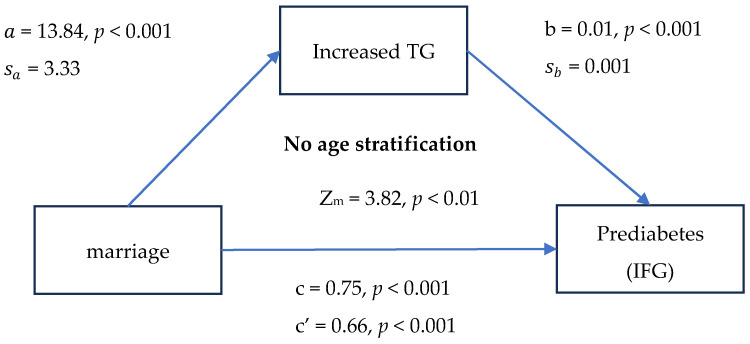

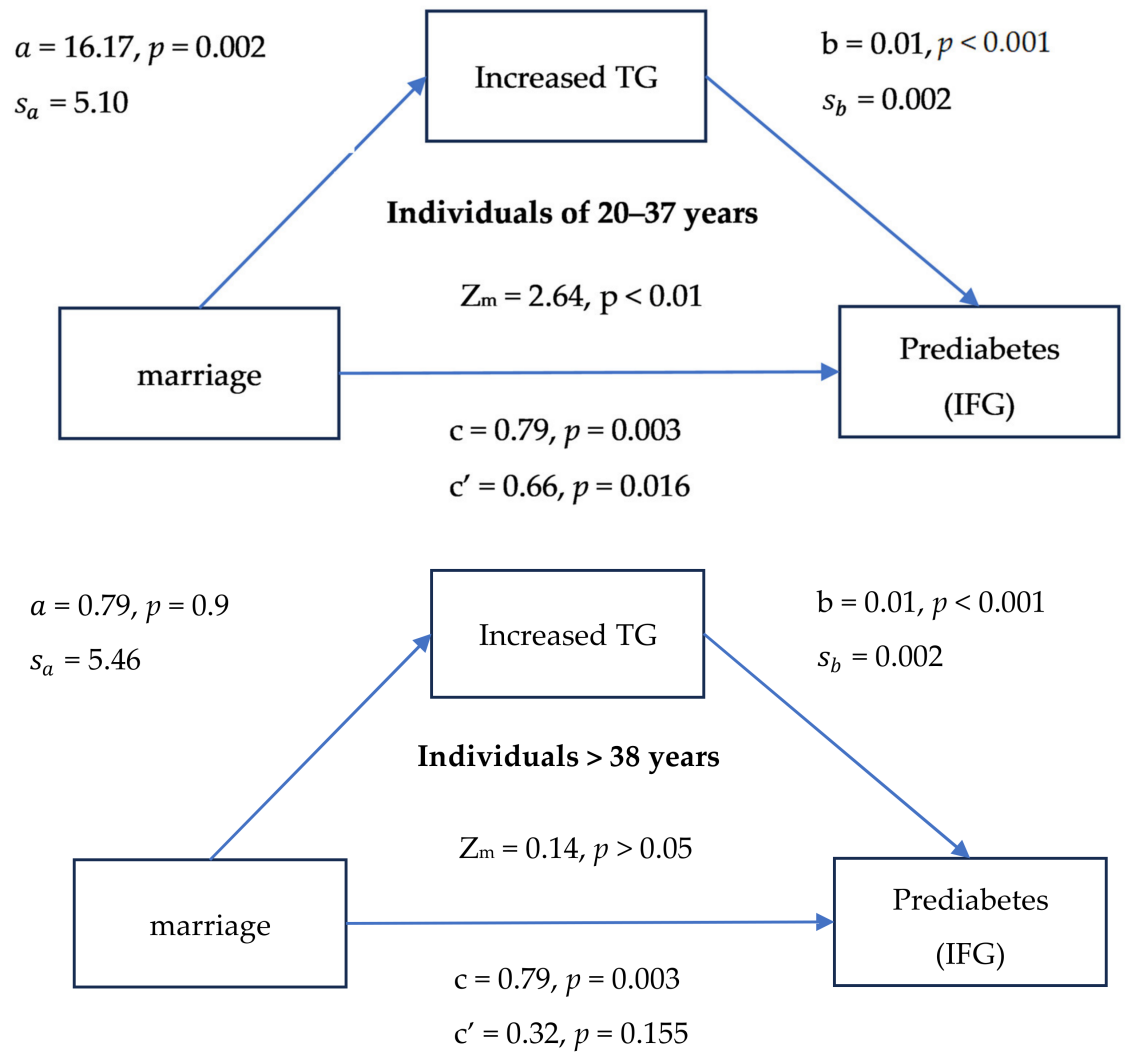
Mediation model of marriage, IFG, and mediating factor triglycerides. a is the linear regression coefficient of married against increased triglycerides; b is the regression coefficient of increased triglycerides against prediabetes in the logistic regression model; $s_{a}$ and $s_{b}$ are the standard deviations of a and b, respectively; c is the logistic regression coefficient of married against IFG in the absence of adjusted variables; and c’ is the logistic regression coefficient of married against IFG in the presence of a mediating factor—increased TG.

Figure 1 shows that the first- and second-stage effects were significant for no age stratification and the group aged 20–37 years (*p* < 0.001; *p* = 0.002/*p* < 0.001 for both) but not the group aged > 38 years. Further statistical tests for the presence of a mediating effect used a formula in Iacobucci (2012) [33] and showed that elevated TG is a mediating factor for the married group and increases the risk of prediabetes with no age stratification and for the group aged 20–37 years (Z_m_ = 3.82; Z_m_ = 2.64, *p* < 0.05 for both).

The results confirmed H2 that increased TG is a mediating factor by which being married increases the IFG risk. The effect was not significant for the group aged > 38 years.

## 4. Discussion

We confirmed that being married in H1 is an independent risk factor for IFG. Nevertheless, the effect of marriage status on IFG in the group > 38 years of age was not significant (OR = 1.43, *p* > 0.05).

Prediabetes prevalence in adults aged ≥ 20 years in the United States (National Health and Nutrition Examination Survey 2015–2016) was 43.5% [34]. Prediabetes prevalence in adults in Taiwan aged ≥ 18 years (National Nutrition and Health Status Change Survey Results Report 2017–2020) was 25.5% [35]. Compared with that of healthcare workers in our study, the proportion of individuals with IFG was 21.17%, close to Taiwan’s general prevalence. Next, we deeply examine confounders of prediabetes, including sex, age, marital status, overweight or obesity status, chronic diseases, and TG levels.

The M_1_ model of Table 2 with no age stratification showed no sex difference in IFG risk (female vs. male, OR = 0.81, *p* < 0.05). A cross-sectional study in England from 2003 to 2011 showed that the odds ratios of prediabetes for males vs. females were 0.96 (95% CI: 0.83, 1.12) and 1.07 (95% CI: 0.91, 1.25), respectively [36]. Despite differences in race and adjusted variables, the findings across studies remain consistent.

A study in Texas, 2004–2017, showed that the odds ratio for prediabetes was 1.7 for individuals aged 40–64 years and 3.1 for those aged >65 years, compared with those aged 18–39 years (*p* < 0.05 for both) [37]. We found that the IFG risk for the group >38 years was 2.17 times greater than that for those aged 20–37 years (Table 2, M_0_, OR = 2.17, 95% CI: 1.59, 2.95). In summary, middle-aged and older participants had a greater risk of developing prediabetes than the younger participants.

Obesity is associated with a greater risk for incident type 2 diabetes [5] and may be linked to prediabetes incidence or risk. For instance, a study of prediabetes and diabetes in adults aged 25–64 years in the Czech Republic showed that the prevalence of prediabetes for overweight and obese was 1.419 (95% CI: 1.007, 1.200) and 2.401 times (95% CI: 1.666, 3.461) greater, respectively, than that for healthy individuals [38]. We showed that in the M_0_ and M_1_ models for all age stratification, prediabetes risk for obese was 2.95–4.54 times than for healthy weight (Table 2, OR = 2.95~4.54, *p* < 0.05 for all). Therefore, maintaining a healthy weight can effectively prevent the occurrence of prediabetes.

Higher glucose levels and diabetes are associated with daily chronic pain in adults [39,40]. Individuals with diabetes/prediabetes often experience chronic pain symptoms in the lower limbs, back, and neck [4]. We also found a close relationship between prediabetes and chronic pain. For example, the participants with IFG more commonly reported neck and shoulder pain than those without IFG with no age stratification, regardless of the M_0_ or M_1_ model (Table 2, OR = 1.23, 95% CI: 1.07, 1.43; OR = 1.19, 95% CI: 1.02–1.40), particularly in the group aged 20–37 years (OR = 1.30, 95% CI: 1.03, 1.65; 1.31, 95% CI: 1.01, 1.69). This finding supports the literature. Notably, chronic pain can pose a significant health risk during physical activity and in dietary habits. These lifestyle changes may increase the risk of developing type 2 diabetes [41].

A study of adults aged 20–39 years showed a greater proportion of married than single males and females had prediabetes [42]. A study among females in Saudi Arabia showed that the prediabetes risk in those who were married was 2.51 times greater (OR = 2.51, 95% CI: 1.61, 3.93) than for their single counterparts [19]. As shown in Table 2, in this study, IFG risk for married was 2.12 (M_0_, OR = 2.12, 95% CI: 1.56, 2.86) and 1.65 (M_1_, OR = 1.65, 95% CI: 1.14, 2.38) times greater than for not married. Nevertheless, another study showed that diabetes was less prevalent among married than unmarried, widowed, or divorced adults [17]. The effect of marriage on diabetes development in adults with prediabetes might be complex and staged by age. For instance, diabetes incidence in males increased with their income, and the opposite was observed for females [17]. We showed that the effect of marriage on IFG was significant for the group aged 20–37 years (M_1_ in Table 2, OR = 1.89, 95% CI: 1.08, 3.33) but not for those aged >38 years. (M_1_ in Table 2, OR = 1.43, 95% CI: 0.91, 2.26). Therefore, whether being married is a protective factor against prediabetes or diabetes in middle-aged and older adults requires further research.

Studies have shown that dieting habits can change after marriage because spouses eat together [13,14,15], which can increase the risk of diabetes [16] and prediabetes. TG levels in men and women were associated with the percentage of calories from fats [25]. In overnutrition, hepatic fatty acid levels are increased due to enhanced lipolysis within adipocytes, leading to increased circulating levels of fatty acids in the bloodstream and increased hepatic de novo lipogenesis. Excess fatty acids cannot be consumed by oxidative pathways; fatty acids are instead directed toward the synthesis of TG, leading to increased hepatic TG storage and very low-density lipoprotein overproduction [23]. Excess TG is packaged into chylomicrons, secreted into the lymphatic system, ultimately reaches the plasma, and is taken up by muscle and adipose tissue [24]. Therefore, triglycerides are closely related to fat intake. Notably, in the group aged 20–37 years, TG levels were higher for the married than the unmarried group (Figure 1, a = 13.84, *p* < 0.001; a = 16.17, *p* = 0.002). One possible explanation is that the dietary habits of married and unmarried adults differ, particularly in higher fat consumption.

Similar to eating habits, TG levels are associated with the risk of type 2 diabetes [43,44,45]. TG might also be associated with an increased risk of prediabetes. For instance, TG may directly contribute to disorders of glucose metabolism [46]. A study of prediabetes in Mexican Americans showed that high TG levels were associated with the risk of prediabetes, particularly in males aged 18–39 years (OR = 1.003, 95% CI: 1.001, 1.005) [37]. We found that increased TG levels increased the IFG risk in all age stratifications (Table 2, OR = 1.003–1.01, *p* < 0.05), indicating the close relationship between TG and IFG regardless of physiology or epidemiology.

The mediation models in Figure 1 showed higher TG levels for married than unmarried adults, which further increased the risk of prediabetes for the former, especially those aged 20–37 years. A study of eating habits in Australia showed that younger people eat more carbohydrates and meat than older adults do [47]. Other studies have shown that the greater TG level in older than younger people [48,49] can be attributed to delayed postprandial TG clearance [50]. Middle-aged and older adults sustain a higher TG level than young adults, regardless of marital status, weakening the effect of marriage on TG levels increasing with age. We also found no significant difference in TG levels between married and unmarried adults >38 years of age (Figure 1, a = 0.79, *p* = 0.9). TG levels were greater for married adults aged 20–37 years than for unmarried adults (Figure 1, a = 16.17, *p* = 0.002).

Age, overweight/obesity status, pain in the neck and shoulders, and increased TG levels are independent risk factors for prediabetes and are highly consistent with the risk factors for diabetes, indicating that prediabetes is an important period for diabetes prevention. Because prediabetes can be reversed [3], increased investment in resources to help individuals with prediabetes improve their living habits, maintain healthy weight, relieve pain, and provide healthy eating information should increase. Moreover, increased attention to the eating habits of young couples and additional healthy eating education to reduce fat intake are necessary.

Some studies have suggested that marriage is not an independent risk factor for prediabetes [19,42]. This finding differs from that of our study and might result from different sample sources (our focus was healthcare workers) and adjustment variables (e.g., we ignored physical activity and family history of diabetes). Specifically, our participants were mainly healthcare workers from a single medical institution, excluding the effects of other occupations on prediabetes (e.g., working time, rotation shifts, work environment conditions). In addition, we did not use an accurate physical activity questionnaire (e.g., Global Physical Activity Questionnaire) to measure the amount of exercise. According to a study of prediabetes and diabetes in South Korea [42], low physical activity in adults was a predictor of prediabetes, which might explain the partial effect of marriage on prediabetes.

A study of 4190 young adults aged 20–39 years in South Korea showed that the prevalence of prediabetes according to fasting blood glucose and hemoglobin A1c standards was 21.2% [42]. We used fasting blood glucose levels between 100 and 125 mg/dL to indicate prediabetes, and the prevalence of prediabetes in the group aged 20–37 years was 15.01% (=80/533). This might be because individuals whose hemoglobin A1c < 5.7% were excluded from prediabetes, leading us to underestimate the proportion of those with prediabetes. A study showed that solely using fasting glucose levels may result in an underestimation of diabetes and prediabetes [51]. However, there was a significant positive correlation between HbA1c and FBG (r2 = 0.713, *p* < 0.05) [52], and we used the FBG level because the determination of the association between prediabetes and marriage-related prediabetes is suitable.

Requirements for sample size calculation based on the prevalence rate include population size, expected prevalence, confidence level (usually 95%), and accepted deviation from the expected prevalence (commonly 5%) [53]. The population size in this study was 2369 individuals. The expected prevalence was 25.5% according to the prediabetes prevalence in adults in Taiwan aged ≥ 18 years [35]. We used Epi Info, developed by the Centers for Disease Control and Prevention, available via the link https://www.cdc.gov/epiinfo/index.html (accessed on 20 August 2024). The calculated result showed that the required sample size for this study is 260 individuals. Our valid sample size is 1039, meeting the criteria.

We further observe the odds ratio of the M_1_ model in Table 2 and find that the values for doctors, nurses, and technicians are all less than 1 (the reference variable is administrative staff). Although the results are not statistically significant, it still implies that the chance of administrative staff suffering from prediabetes may be higher than that of participants in other medical professional fields. Since our study did not collect data on diabetes prevention knowledge, future research could design new questionnaires to gather relevant information. This would help confirm whether this phenomenon truly exists and whether it is due to differences in diabetes prevention concepts.

A Portuguese study on adolescent obesity showed that people who eat a lot of meat have a higher prevalence of hypertension and significant TG levels [54] supplementary. Based on our study, we found that triglycerides play an important role in the development of prediabetes in young couples. In terms of diabetes prevention strategies, we cannot help but think about a question: Does this development model also apply to teenagers? Do teenagers living in different environments also have a higher incidence of prediabetes due to dietary and cultural differences? This issue will be further explored in future research.

Our study used a self-report questionnaire in the hope of obtaining a larger sample size, but this may raise some questions about the quality of the responses. Therefore, we used regression statistics to mitigate these effects by controlling for variables. Furthermore, our participants were all healthcare workers from the same institution, so whether the results we obtained are applicable to other professions requires further research. The disparity in the ratio of men to women is also a concern. Although we adjusted for gender factors, this may still have impacted the research results. A more rigorous stratified sampling method should be adopted for future investigations.

## 5. Conclusions

Increasing age, marital status, being overweight or obese, neck and shoulder pain, and high TG levels are independent risk factors for prediabetes among healthcare workers. Additionally, married adults had higher TG levels were higher in married than in unmarried adults, elevating prediabetes risk, particularly in those aged 20–37 years. Consequently, the dietary habits of young couples warrant increased attention. While starting a family and raising children, people should also pay more attention to the dietary health of both their family and themselves to reduce the risk of prediabetes. Our results link marital status to diabetes and will support advances in diabetes prevention. The same research method can be extended to other occupations and even younger groups, such as teenagers, to discover risk factors that may have been ignored in the past. This will make a greater contribution to diabetes prevention.

## Figures and Tables

**Table 1:** Demographic and survey variables for 1039 participants and stratified analysis of prediabetes (IFG) by age.

Surveyed Variable	N (%)	IFG (Individuals = 220; 21.17%)							
		No age Stratification		Aged 20–37 Years			Aged > 38 Years		
		n (%)/m ± SD	*p*	N’	n (%)/m ± SD	*p*	N’	n (%)/m ± SD	*p*
Sex									
Female	885 (85.18)	173 (19.55)	0.004 ^a^	452	61 (13.05)	0.028 ^a^	433	112 (25.87)	0.034 ^a^
Male	154 (14.82)	47 (30.52)		81	19 (23.46)		73	28 (38.36)	
Age									
20–37 years	533 (51.30)	80 (15.01)	<0.001 ^a^		-	-		-	-
>38 years	506 (48.70)	140 (27.76)			-	-		-	-
Married									
Yes	453 (43.60)	128 (28.26)	<0.001 ^a^	112	27 (24.11)	0.004 ^a^	341	101 (29.62)	0.169 ^a^
Other	586 (56.40)	92 (15.70)		421	53 (12.59)		165	39 (23.64)	
Raising children									
Yes	395 (38.02)	108 (27.34)	<0.001 ^a^	82	21 (25.61)	0.007 ^a^	313	87 (27.80)	1.00 ^a^
No	644 (61.98)	112 (17.39)		451	59 (13.08)		193	53 (27.46)	
Fostering parents									
Yes	537 (51.68)	123 (22.91)	0.172 ^b^	244	38 (15.57)	0.808 ^a^	293	85 (29.01)	0.481 ^a^
No	502 (48.32)	97 (19.32)		289	42 (14.53)		213	55 (25.82)	
Drinking coffee habits									
Never	169 (16.27)	31 (18.34)	0.111 ^b^	127	19 (14.96)	0.392 ^b^	42	12 (28.57)	0.972 ^b^
Occasionally	456 (43.89)	85 (18.64)		263	33 (12.55)		193	52 (26.94)	
One cup per day	355 (34.17)	87 (24.51)		126	24 (19.05)		229	63 (27.51)	
Two cups per day	45 (4.33)	12 (26.67)		14	3 (21.43)		31	9 (29.03)	
At least two cups per day	14 (1.35)	5 (35.71)		3	1 (33.33)		11	4 (36.36)	
Alcohol use in past month									
Never	653 (62.85)	137 (20.98)	0.864 ^b^	319	43 (13.48)	0.381 ^b^	334	94 (28.14)	0.246 ^b^
Occasionally	383 (36.86)	82 (21.41)		212	37 (17.45)		171	45 (26.32)	
Drinking every day	3 (0.29)	1 (33.33)		2	0 (0)		1	1 (100)	
Sleeping time every day									
<5 h	47 (4.52)	9 (19.15)	0.440 ^b^	26	4 (15.38)	0.307 ^b^	21	5 (23.81)	0.568 ^b^
5–6 h	377 (36.28)	85 (22.55)		209	40 (19.14)		168	45 (26.79)	
6–7 h	444 (42.73)	98 (22.07)		199	24 (12.06)		245	74 (30.20)	
7–8 h	143 (13.76)	25 (17.48)		81	10 (12.35)		62	15 (24.19)	
>8 h	28 (2.69)	3 (10.71)		18	2 (11.11)		10	1 (10.00)	
Exercise habits									
Never	65 (6.26)	9 (13.85)	0.348 ^b^	29	4 (13.79)	0.924 ^b^	36	5 (13.89)	0.195 ^b^
Less than once monthly	249 (23.97)	51 (20.48)		147	25 (17.01)		102	26 (25.49)	
At least once monthly	191 (18.38)	37 (19.37)		108	17 (15.74)		83	20 (24.10)	
At least once weekly	464 (44.66)	104 (22.41)		228	31 (13.60)		236	73 (30.93)	
At least once daily	70 (6.74)	19 (27.14)		21	3 (14.29)		49	16 (32.65)	
Body weight									
Underweight	88 (8.47)	7 (7.95)	<0.001 ^b^	69	6 (8.70)	<0.001 ^b^	19	1 (5.26)	<0.001 ^b^
Healthy weight	616 (59.29)	97 (15.75)		319	38 (11.91)		297	59 (19.87)	
Overweight	192 (18.48)	55 (28.65)		70	11 (15.71)		122	44 (36.07)	
Obesity	143 (13.76)	61 (42.66)		75	25 (33.33)		68	36 (52.94)	
Chronic diseases (excluding diabetes)									
Yes	389 (37.44)	99 (25.45)	0.009 ^a^	164	31 (18.90)	0.114 ^a^	225	68 (30.22)	0.272 ^a^
No	650 (62.56)	121 (18.62)		369	49 (13.28)		281	72 (25.62)	
Profession fields									
Physician	121 (11.65)	30 (24.79)	0.005 ^b^	61	13 (21.31)	0.294 ^b^	60	17 (28.33)	0.043 ^b^
Nurses	475 (45.72)	81 (17.05)		272	34 (12.50)		203	47 (23.15)	
Technical staff	102 (9.82)	18 (17.65)		47	7 (14.89)		55	11 (20.00)	
Administration staff or others	341 (32.82)	91 (26.69)		153	26 (16.99)		188	65 (34.57)	
Work time									
Work time daily (hrs.)	1039	8.52 (0.88)	-	533	8.56 (0.83)	-	506	8.47 (0.92)	-
Shift work									
Irregular shift	158 (15.21)	27 (17.09)	0.008 ^b^	104	12 (11.54)	0.139 ^b^	54	15 (27.78)	0.830 ^b^
Regular shift	132 (12.70)	18 (13.64)		97	10 (10.31)		35	8 (22.86)	
Night shift	133 (12.80)	23 (17.29)		87	12 (13.79)		46	11 (23.91)	
Day shift	616 (59.29)	152 (24.68)		245	46 (18.78)		371	106 (28.57)	
Education degree									
PhD.	9 (0.87)	4 (44.44)	0.013 ^b^	1	0 (0)	0.004 ^b^	8	4 (50)	0.487 ^b^
Master	137 (13.19)	33 (24.09)		49	9 (18.37)		88	24 (27.27)	
Bachelor	856 (82.39)	169 (19.74)		477	67 (14.05)		379	102 (26.91)	
Others	37 (3.56)	14 (37.84)		6	4 (66.67)		31	10 (32.26)	
Burnout									
Personal burnout	1039	39.26 ± 19.75	-	533	40.87 ± 19.90	-	506	37.56 ± 19.47	-
The frequency on Musculoskeletal pain									
Neck and shoulder pain	1039	0.003 ± 0.93	-	533	−0.027 ± 0.84	-	506	0.029 ± 1.00	-
Pain in both ankles	1039	0.003 ± 0.81	-	533	−0.056 ± 0.44	-	506	0.059 ± 1.06	-
Health check									
FBG (mg/dL)	1039	93.49 ± 8.60	-	533	91.89 ± 7.62	-	506	95.18 ± 9.25	-
TG (mg/dL)	1039	74.03 ± 53.63	-	533	65.82 ± 48.36	-	506	82.68 ± 57.47	-

N: individuals comprising all participants; N’: individuals in age group; n: individuals with prediabetes; m: mean value; SD: standard deviation; ^a^: Fisher exact test; ^b^: chi-square test; *p*: *p* value.

**Table 2:** Odds ratio of surveyed variables against IFG for stratified analysis of age grouping.

	OR (95% CI)					
	No age Stratification		20–37 Years		>38 Years	
Surveyed Variable	M_0_	M_1_	M_0_	M_1_	M_0_	M_1_
**Main effect**						
Married ^3^	**2.12 (1.56, 2.86)**	**1.65 (1.14, 2.38)**	**2.21 (1.31, 3.71)**	**1.89 (1.08, 3.33)**	1.36 (0.89, 2.09)	1.43 (0.91, 2.26)
TG	**1.01 (1.006, 1.011)**	**1.004 (1.00, 1.01)**	**1.01 (1.005, 1.015)**	**1.01 (1.00, 1.01)**	**1.01 (1.003, 1.01)**	**1.003 (1.00, 1.01)**
**Confounders**						
Female ^1^	**0.55 (0.38, 0.81)**	0.81 (0.52, 1.28)	**0.51 (0.29, 0.91)**	0.84 (0.43, 1.62)	**0.56 (0.33, 0.94)**	0.91 (0.50, 1.64)
>38 years ^2^	**2.17 (1.59, 2.95)**	1.37 (0.94, 1.99)		-		
Raising children ^4^	**1.79 (1.32, 2.42)**	-	**2.29 (1.30, 4.03)**	-	1.02 (0.68, 1.52)	-
Fostering parents ^5^	1.24 (0.92, 1.67)	-	1.09 (0.67, 1.75)	-	1.17 (0.79, 1.75)	-
Drinking coffee every day ^6^	**1.47 (1.09, 1.99)**	1.11 (0.79, 1.56)	1.58 (0.95, 2.62)	-	1.04 (0.70, 1.54)	-
Ever alcohol use in a month ^7^	1.03 (0.76, 1.40)	-	1.34 (0.83, 2.17)	-	0.93 (0.62, 1.41)	-
Sleeping time less than 6 h ^8^	1.11 (0.82, 1.49)	-	**1.68 (1.04, 2.71)**	1.47 (0.88, 2.44)	0.91 (0.61, 1.36)	-
Exercise at least once weekly ^9^	1.26 (0.93, 1.70)	-	0.82 (0.51, 1.32)	-	**1.51 (1.01, 2.26)**	1.47 (0.95, 2.28)
Underweight ^10^	0.46 (0.21, 1.03)	0.70 (0.31, 1.59)	0.70 (0.29, 1.74)	0.91 (0.36, 2.29)	0.22 (0.03, 1.71)	0.31 (0.04, 2.39)
Overweight ^10^	**2.15 (1.47, 3.14)**	**1.66 (1.10, 2.50)**	1.38 (0.67, 2.85)	1.14 (0.53, 2.45)	**2.28 (1.43, 3.63)**	**2.08 (1.27, 3.43)**
Obesity ^10^	**3.98 (2.68, 5.91)**	**3.45 (2.22, 5.37)**	**3.70 (2.06, 6.65)**	**2.95 (1.49, 5.83)**	**4.54 (2.61, 7.91)**	**4.30 (2.38, 7.79)**
Chronic diseases (excluded diabetes) ^11^	**1.49 (1.10, 2.02)**	1.35 (0.97, 1.88)	1.52 (0.93, 2.49)	-	1.26 (0.85, 1.86)	-
Physician ^12^	0.91 (0.56, 1.46)	0.83 (0.49, 1.40)	1.32 (0.63, 2.78)	-	0.75 (0.40, 1.41)	0.69 (0.35, 1.36)
Nurse ^12^	**0.57 (0.40, 0.79)**	0.74 (0.50, 1.10)	0.70 (0.40, 1.22)	-	**0.57 (0.37, 0.89)**	0.62 (0.38, 1.01)
Technical staff ^12^	0.59 (0.34, 1.03)	0.58 (0.32, 1.06)	0.86 (0.35, 2.12)	-	**0.47 (0.23, 0.98)**	0.46 (0.22, 1.00)
Work time every day	0.97 (0.82, 1.16)	-	1.10 (0.83, 1.45)	-	0.94 (0.75, 1.17)	-
Irregular shift work ^12^	**0.63 (0.40, 0.99)**	0.84 (0.51, 1.40)	0.56 (0.29, 1.12)	-	0.96 (0.51, 1.82)	-
Regular shift work ^13^	**0.48 (0.28, 0.82)**	0.56 (0.31, 1.01)	0.50 (0.24, 1.03)	-	0.74 (0.33, 1.68)	-
Night shift work ^13^	0.64 (0.39, 1.04)	0.94 (0.54, 1.64)	0.69 (0.35, 1.38)	-	0.79 (0.39, 1.60)	-
Master or PhD. ^14^	1.32 (0.88, 1.98)	-	1.27 (0.59, 2.74)		1.10 (0.67, 1.79)	-
Personal burnout	1.00 (0.99, 1.01)	-	1.01 (0.99, 1.02)	-	1.00 (0.99, 1.01)	-
Neck and shoulder pain	**1.23 (1.07, 1.43)**	**1.19 (1.02, 1.40)**	**1.30 (1.03, 1.65)**	**1.31 (1.01, 1.69)**	1.18 (0.98, 1.43)	-
Pain in both	1.11 (0.95, 1.31)	-	0.59 (0.25, 1.38)	-	1.12 (0.95, 1.33)	-

OR: odds ratio; M_0_: binary logistic regression model; M_1_: multiple logistic regression model in the presence of adjusted variables; ^1^: reference variable, male; ^2^: reference variable, 20–37 y; ^3^: reference variable, unmarried; ^4^: reference variable, not raising children; ^5^: reference variable: not fostering parents; ^6^: reference variable, never or occasionally drinking coffee; ^7^: reference variable, never uses alcohol; ^8^: reference variable, sleeping time more than 6 h; ^9^: reference variable, less than one time weekly; ^10^: reference variable, healthy weight; ^11^: reference variable, without chronic diseases; ^12^: reference variable, administration staff; ^13^: reference variable, day shift work; ^14^: reference variable, bachelor and other. Bold font indicates statistical significance (*p* < 0.05).

## Data Availability

The datasets used and/or analyzed during the current study are available from the corresponding author on reasonable request.

## Supplementary Materials

The online version contains supplementary material available at: https://www.mdpi.com/article/10.3390/medicina60091418/s1, Table S1: Musculoskeletal pain sites and factor analysis of the Nordic Musculoskeletal Questionnaire.
