# Supplementary material for: Relationship between Marriage and Prediabetes among Healthcare Workers: Mediating Effect of Triglycerides
Source: Medicina (Kaunas). 2024 Aug 30;60(9):1418. doi: 10.3390/medicina60091418 (PMC11434444; doi:10.3390/medicina60091418)
Supplement: Supplementary file 1 [file medicina-60-01418-s001.zip › medicina-3173077-supplementary.pdf]

Supplementary Information Table S1. Musculoskeletal pain sites and factor analysis of the Nordic Musculoskeletal Questionnaire.

Musculoskeletal pain sites	Multiplied product [1]		Factor loading [2]	
	Mean	SD	Factor 1	Factor 2
**Neck** [3]	517.85	1,010.84	**0.33**	−0.01
**Left shoulder** [3]	329.98	933.66	**0.32**	−0.08
**Right shoulder** [3]	348.87	905.24	**0.30**	0.00
Upper back	270.40	906.58	0.15	−0.03
Waist or lower back	538.14	1,180.10	0.08	−0.04
Left elbow	57.65	440.66	−0.06	−0.14
Right elbow	107.51	624.74	−0.01	−0.01
Left wrist	89.17	537.78	−0.02	0.07
Right wrist	196.87	754.77	−0.01	0.18
Left hip/thigh/buttock	84.84	664.35	−0.03	−0.18
Right hip/thigh/buttock	88.07	615.27	−0.03	0.02
Left knee	91.48	575.78	0.00	−0.05
Right knee	74.49	483.41	−0.06	−0.01
**Left ankle** [3]	56.79	459.45	−0.02	**0.57**
**Right ankle** [3]	43.19	354.38	−0.02	**0.37**
Eigenvalues			6.06	1.83
Accumulation of explained variation (%)			63.87	83.16

[1], It is a product of frequency score multiplied by serious degree score; [2], The factor loading of eigenvalues more than 1; [3], Bold fonts represent pain sites with relatively large factor loading.
